# Development and evaluation of a Smartphone-enabled, caregiver-supported educational intervention for management of physical disabilities following stroke in India: protocol for a formative research study

**DOI:** 10.1136/bmjinnov-2015-000042

**Published:** 2015-07

**Authors:** K Sureshkumar, G V S Murthy, Sanjay Kinra, Shifalika Goenka, Hannah Kuper

**Affiliations:** 1 International Centre for Evidence in Disability, Department of Clinical Research, London School of Hygiene and Tropical Medicine, London, UK; 2 Department of Non-Communicable Disease, Epidemiology, London School of Hygiene and Tropical Medicine, London, UK; 3 Indian Institute of Public Health – Delhi

**Keywords:** mHealth, Geriatrics, Neurology, Medical Apps, Global Health

## Abstract

The incidence and prevalence of stroke in India has reached epidemic proportions. The growing magnitude of disability in patients with stroke in India poses a major public health challenge. Given the nature of the condition, affected individuals often become disabled with profound effects on their quality of life. The availability of rehabilitation services for people with disabilities is inadequate in India. Rehabilitation services are usually offered by private hospitals located in urban areas and many stroke survivors, especially those who are poor or live in rural areas, cannot afford to pay for, or do not have access to, such services. Thus, identification of cost-effective ways to rehabilitate people with stroke-related disability is an important challenge. Educational interventions in stroke rehabilitation can assist stroke survivors to make informed decisions regarding their on-going treatment and to self-manage their condition with support from their caregivers. Although educational interventions have been shown to improve patient knowledge for self-management of stroke, an optimal format for the intervention has not as yet been established, particularly in low- and middle-income countries. This formative research study aims to systematically develop an educational intervention for management of post-stroke disability for stroke survivors in India, and evaluate the feasibility and acceptability of delivering the intervention using Smartphones and with caregiver support. The research study will be conducted in Chennai, India, and will be organised in three different phases. *Phase 1:* Development of the intervention. *Phase 2:* Field testing and finalising the intervention. *Phase 3:* Piloting of the intervention and assessment of feasibility and acceptability. A mixed-methods approach will be used to develop and evaluate the intervention. If successful, it will help realise the potential of using Smartphone-enabled, carer-supported educational intervention to bridge the gaps in service access for rehabilitation of individuals with stroke-related disability in India. The proposed research will also provide valuable information for clinicians and policymakers.

## Background

Stroke is a major global public health problem. According to the Global Burden of Disease (GBD) study in 2010, stroke is the second leading cause of death worldwide.^1^ A person experiences a stroke when a blood clot blocks a blood vessel in the brain or a vessel that supplies it, or when there is bleeding in the brain. The interruption of blood supply to the brain reduces the supply of oxygen and nutrients to it, causing injury and death of brain tissue.^2^ This brain damage may subsequently result in long-term disability or death of the affected individual.^2^

Stroke is associated with a wide variety of sensory-motor, cognitive-perceptual and behavioural impairments.^3^ The effects of stroke will depend on the site of the brain lesion and severity of brain damage.^4^ In addition to the primary impairments following a stroke, secondary complications of stroke can also hamper the recovery process.^5^ The prognosis in stroke depends on the degree of primary impairments and secondary complications.^4^

Disability is an umbrella term, covering impairments, activity limitations and participation restrictions.^6^ Impairment following stroke may present as physical, mental or cognitive. Stroke impairment might limit the ability of the stroke survivor to independently perform his or her daily living activities (eg, difficulties in walking or communicating).^6^ Consequently, it might also restrict effective participation of the stroke survivor in his/her family and social roles.^7^ Disability following stroke depends on the degree of impairment (physical, mental, cognitive) as well as the personal and contextual environment of the affected individual. Most often, stroke survivors become disabled with profound effect on their quality of life.^8^

The impact of disability following a stroke also affects the family of the stroke survivor.^9^ Adapting to the new role of a carer and adjusting to the sudden impact of stroke can be highly stressful for family members.^10^ The demand on caregivers increases tremendously, especially if the stroke survivor experiences severe disability.^11^ The rehabilitation needs of stroke survivors and their family will vary extensively based on the degree of impairment and the context in which they experience a stroke (eg, accessibility to stroke services, family support, etc).^7^ ^9–12^

## Rehabilitation needs of stroke survivors in India

Evidence from a literature review suggests that India is experiencing a silent epidemic of stroke.^13^ Prevalence rate of stroke in India is estimated to range from 84 to 262/100 000 in rural areas and 334 to 424/100 000 in urban areas. The incidence rate is 119–145/100 000 based on recent population based studies.^14^ The incidence and prevalence of stroke was observed to be higher in India, compared to the incidence and prevalence of stroke in high-income countries (HICs).^13^ Unlike HICs, there is a dearth of information about the rehabilitation needs of people with disabilities following stroke in India.^14^ People with disabilities in general encounter tremendous environmental barriers in accessing rehabilitation services in India.^15^ Lack of policy initiatives for rehabilitation, inadequate rehabilitation resources and health professionals, lack of an accessible environment and stigma are some of the major barriers that persons with disability experience in India.^16^ Taking into account the disability after stroke and the existing environmental barriers to rehabilitation, the needs of stroke survivors in India are expected to be substantial and diverse.

## Rehabilitation services in India

Rehabilitation services in India are usually hospital-based and driven predominately by physiotherapists. Therapy inputs from other health professionals, such as occupational therapists and speech therapists, are hardly available to patients with stroke.^17^ A recent study undertaken by the Public Health Foundation of India (PHFI) for the Ministry of Health and Family Welfare (MoHFW) has indicated a supply-demand gap of about 6 500 000 allied health professionals in India.^18^ Even the information needs of patients with stroke and their primary caregivers to self-manage their problems following stroke remain largely unfulfilled.^7^ Provision of rehabilitation services in India are usually limited to specialised hospitals located in urban areas, and many people, especially those who are poor or who live in rural areas cannot afford to pay for, or have limited access to, such services.^18^ When patients and caregivers travel long distances to obtain rehabilitation services, there is a huge financial implication and opportunity cost involved in accessing these services.^7^ Although the number of private rehabilitation facilities in India has increased, these are only accessed by a minuscule proportion of the country's vast population.^19^ Owing to these reasons, most people with disabilities following stroke do not have access to rehabilitation services in India.^20^ The existing barriers to rehabilitation suggest that the rehabilitation needs of the stroke survivors in India remain largely unmet.

Given the context, it is imperative that stroke survivors and their caregivers are educated about stroke and the ways to manage post-stroke disability on their own. Educational intervention could assist stroke survivors and their families to access support services and to make informed decisions regarding their care.^21^ ^22^ Educational interventions were found to improve patients’ and carers’ knowledge on the self-management of stroke.^23^

A chronic condition such as stroke requires uninterrupted therapeutic care and constant monitoring during the entire continuum of recovery.^23^ In the absence of any organised stroke care services and with the limited resources for rehabilitation, a Smartphone-enabled educational intervention for management of disability could be a strategy to meet the substantial rehabilitation needs of stroke survivors in India.^24^ The evidence concerning the use of Smartphones in chronic disease care in India is finally emerging and the use of Smartphones in interventions to combat diseases such as diabetes, hypertension and cardiovascular diseases, is progressively being investigated.^25^ In some HICs, Smartphones are used to create awareness about the warning signs of stroke^26^ and also to aid rehabilitation of language and communication impairments following stroke.^27^ Adoption of this strategy could possibly reduce the barriers to access and availability of stroke rehabilitation services. It could also aid in efficient and sustained monitoring of patient progress throughout the continuum of care. Thus, this study seeks to develop and evaluate a Smartphone-enabled carer-supported educational intervention for management of physical disabilities following stroke in India.

## Overall aim and strategy of the study

The aim of this study is to develop and evaluate a Smartphone-enabled, carer-supported education programme for stroke survivors in India. The purpose of this formative research is to systematically (conforming to the MRC framework^28^) develop an educational intervention for management of post-stroke disability for stroke survivors in India, and evaluate the feasibility and acceptability of delivering the intervention using Smartphones and with caregiver support.

**Figure 1:**
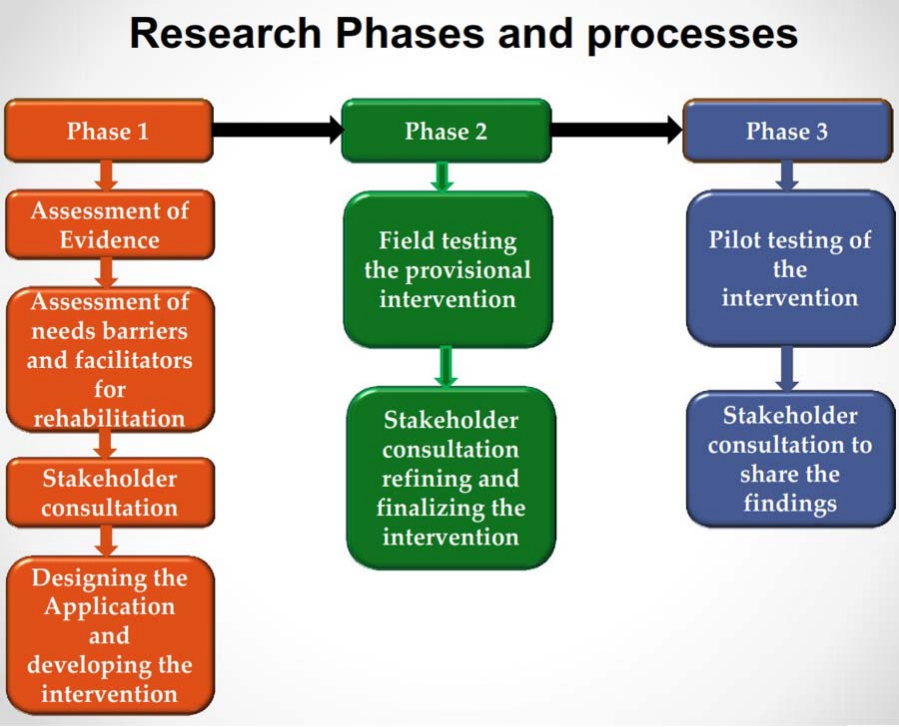
Flow chart of the processes and activities of the research phases.

This study will be conducted in three phases: (1) development of the intervention; (2) pre-testing of the intervention and stakeholder consultation; and (3) piloting of the intervention, and assessment of feasibility and acceptability. Processes and activities involved in each phase of the research study are explained using a flow chart in figure 1, and are described in detail below.

This study will apply mixed research methods in order to collect more comprehensive evidence and have a deeper understanding of the research problem. Mixed methods research encourages the use of multiple worldviews, and is a practical and natural approach to research pertaining to development of a complex intervention.^29^ It is premised on the idea that the use of quantitative and qualitative approaches in combination provides a better understanding of research problems than either approach alone.^30^

## Proposed study design

### Methods: phase 1: Development of the intervention

The overall objective of this phase is to develop a Smartphone-enabled, carer-supported education intervention for stroke survivors to manage their post-stroke disability. Research questions that will be answered in this phase along with the methods are detailed in table 1. At the end of phase I, a provisional Smartphone-enabled educational intervention for management of stroke-related disability will be developed for field testing and refinement. This provisional intervention will encompass the rehabilitation needs of patients with stroke and their caregivers identified in this formative phase.

**Table 1:** Objectives, research questions and methods for phase I

Objectives	Questions	Methods
Explore the experience of the stroke survivors and their caregivers in accessing stroke rehabilitation services	What impact does a stroke cause in the life of a stroke survivor, and his/her family and caregiver? How does an individual affected by stroke and his/her family organise themselves to manage the effects of stroke? What measures will stroke survivors and their caregivers take in order to manage disability following stroke? What is the general understanding of the stroke survivor and his/her caregiver about stroke rehabilitation? What kind of stroke rehabilitation services are generally available to stroke survivors, from where and from whom? At what phase of recovery are these rehabilitation services available to stroke survivors? How do stroke survivors usually access stroke rehabilitation services? What is the general perception of stroke survivors and their caregivers on the quality of available rehabilitation services? What are the difficulties faced by stroke survivors in accessing rehabilitation services? What is the cost of obtaining stroke rehabilitation services and what is the impact of this increased expenditure on the family?	Survey using structured questionnaire and in-depth interviews with stroke survivors and their caregivers
Assess the information needs of patients with stroke and their carers	What information do stroke survivors and the caregivers need to manage disability following a stroke? What kind of information is available to them, from where and from whom? At what point of time or phase of recovery (acute, post-acute, rehab, long-term care), is this information available to them? What is the quality of available information (regarding correctness, relevance, reliability and understandability)?	Survey using structured questionnaire and in-depth interviews with stroke survivors and their caregivers
Assess the rehabilitation needs of patients with stroke	What are the various kinds of disability experienced by stroke survivors following stroke? What are the various rehabilitation needs (physical, psychological, social) of stroke survivors and their caregivers? What kinds of rehabilitation services are required to address the needs of stroke survivors and their caregivers? What kind of rehabilitation services would enable stroke survivors to be functionally independent following stroke?	Survey using structured questionnaire and in-depth interviews with stroke survivors and their caregivers
Assess the barriers and facilitators for rehabilitation of stroke survivors	What are the present policies and programmes for rehabilitation of persons with disabilities especially following stroke? How are these rehabilitation policies and programmes implemented? Are there adequate resources (human resources, material and financial resources) for rehabilitation of stroke survivors? How are these rehabilitation services delivered to stroke survivors? What are the constraints in providing rehabilitation services to stroke survivors? What can be done to address these constraints? What are the facilitating factors for provision of rehabilitation services for stroke survivors?	In-depth interviews with health professionals providing stroke rehabilitation services in hospitals
Assess the experience and comfort of the stroke survivors and their caregivers in using Smartphone technology	Has the stroke survivor and/or caregiver ever used a Smartphone? What was their experience in using Smartphone technology? How long and for what purpose were they using the Smartphone? What abilities are essential/required to use a Smartphone comfortably? Will a Smartphone-enabled carer-supported education programme be useful for stroke survivors? What are the advantages and disadvantages of such interventions?	In-depth interviews with stroke survivors and their caregivers
Recommendations for action	What are the potential rehabilitation strategies to facilitate functioning, participation and independent living among stroke survivors? What are the resources required to implement the rehabilitation strategy? Is the strategy feasible and sustainable? How can the proposed rehabilitation strategy be implemented and made sustainable? What could be the potential barriers/problems for implementation? What are the possible solutions to address the implementation barriers?	In-depth interviews with health professionals providing stroke rehabilitation services in hospitals

### Detailed methods for phase 1

#### Study setting

Participants will be selected from hospitals within Chennai that provide treatment and rehabilitation services for stroke survivors, and that are willing to recruit participants for this phase. Hospitals that could be potential recruitment sites for this phase will be identified and contacted, and permission will be obtained. Chennai, with a population of over 9 million, is the capital city of the Indian state of Tamil Nadu. It is the biggest industrial and commercial centre in South India, and a major cultural, economic and educational centre in the country.

Participant inclusion criteria:

1. Participants with a recently diagnosed stroke (within the previous 6 weeks) as defined by the WHO;^31^
2. Aged ≥18 years;
3. Presenting with minor and moderate stroke (ie, scoring 1–15, according to the National Institute of Health (NIH) stroke scale);^32–34^
4. Discharged from the hospitals (recruitment sites);
5. Residing at home with a primary caregiver.

Exclusion criteria:

1. Stroke survivors with severe communication problems identified using the NIH stroke scale;^32–34^
2. Stroke survivors who cannot provide consent autonomously;
3. Those presenting with severe stroke (ie, scoring >15, according to the NIH stroke scale).^32–34^

#### Survey of study participants using structured questionnaires

From the participants who meet the inclusion criteria, a purposive sample will be selected for the questionnaire survey.

- *Stroke survivors:* 50 participants admitted to hospital and then discharged within the previous 6 weeks.
- *Primary caregivers of the stroke survivors:* 50 participants.

This phase will have a pragmatic approach to participant recruitment. The initial recruitment will include all eligible participants. In the later stage, recruitment will be more focused on the potential subgroups of participants stratified by their age, gender and severity, for gaining a better understanding of their specific experiences and rehabilitation needs.

The purpose of this survey is to identify the various kinds of rehabilitation needs of stroke survivors, and the barriers and facilitators encountered by stroke survivors to access stroke rehabilitation services.

#### Study participants—in-depth interviews

From the participants selected for the survey, a subsample will be selected for in-depth interviews, including:

- 10–15 stroke survivors;
- 10–15 primary caregivers of stroke survivors.

The purpose of the in-depth interviews is to gain detailed understanding of the experiences of the stroke survivors in relation to accessing stroke rehabilitation services and their rehabilitation needs following stroke. Participants will be asked about their experiences of accessing stroke rehabilitation services, their rehabilitation needs, and about the barriers and facilitators to rehabilitation in various domains of their daily life, such as self-care, mobility and home-management; and leisure, social and vocational activities.

In addition, a purposefully selected sample of 8–10 health professionals from different rehabilitation disciplines (eg, rehabilitation medicine, neurology, physiotherapy, occupational therapy, speech therapy) will be selected and interviewed in depth. The purpose is to understand the perspective of the health professionals about provision of stroke rehabilitation services. This will include their understanding about the barriers and facilitators to accessing stroke rehabilitation services, their knowledge about the existing Smartphone-based health interventions, and their attitudes and opinions about the use of a Smartphone enabled, care-supported education programme for domiciliary stroke rehabilitation.

The in-depth interview process will end when the collection of new qualitative information does not shed any further light on the issue under investigation (saturation point). If the interviews with the proposed number of participants do not reach a saturation point, additional interviews will be conducted until saturation.

An investigator will administer the questionnaire verbally and will be conducting the interviews in English or Tamil, whichever is suitable for the respondents. The interviews for stroke survivors and their primary caregivers will take place at their homes. For the health professionals, the in-depth interviews will take place at their respective hospitals. Interviews and discussions will be conducted in a secluded area so that participants’ privacy and confidentiality is assured. All the interviews will be tape recorded.

#### Study tools

Separate questionnaires and topic guides will be developed for stroke survivors, their primary caregivers and health professionals, and pilot-tested before starting the study. The tools will be revised accordingly after the pilot-testing. The questionnaire will predominantly include close-ended questions with scaled responses. The questionnaire will be developed based on the WHO-Disability Assessment Schedule (WHODAS),^35^ and also from tools used in previous studies.^36^ The in-depth interviews will have specific topic guides with open-ended questions and prompts.

#### Informed written consent

All eligible participants will be informed about the study, and written consent will be obtained from those who are willing to participate. Stroke survivors who are discharged from the hospital (within a 6-week window prior to the study) and their caregivers will be identified using the hospital discharge records and contacted over the phone. The purpose and processes of the study will be explained to the participants and consent will be obtained from potential participants in person.

### Analysis

#### Quantitative analysis of questionnaire survey data

Investigators will use STATA V.13.0 (StataCorp 2013. Stata Statistical Software: Release 13. College Station, Texas: StataCorp LP, USA) for analysis of data from the questionnaire schedule. Data will be double entered and compared, to detect and correct any errors that might have occurred during the data entry. The questionnaire schedule will have specific domains of interest. The descriptive frequencies, and 95% CI for each of these domains will be calculated.

### Qualitative analysis of in-depth interviews

#### Transcribing qualitative data

Transcribing will be carried out to produce a written version of the interview. It is a full ‘script’ of the interview.^30^ Hand written interview notes will be documented in detail on the same day in order to avoid losing information. Tape-recorded information from the in-depth interviews of study participants will be fully transcribed verbatim within 3–5 days after the interviews. Consideration will be given to how certain things were communicated, and to the context, feelings and meanings, while transcribing. Punctuation marks and techniques such as underlining, marking with symbols, using upper case lettering, underlining and emboldening during the transcribing process, will be used during analysis. Each transcribed interview will be reviewed as soon as possible and before the next interview in order to incorporate any interesting findings into the next interview, and to explore them further. Investigators will use the framework approach to carry out the qualitative analysis. The transcribed data will be analysed using the following steps:

- Familiarisation with the data;
- Identifying a thematic framework;
- Indexing;
- Charting;
- Mapping and interpretation.

Results of the analysis will inform the design and development of the intervention. The overall framework of the intervention package will be finalised after the formative phase.

#### Triangulation of information—stakeholder participatory workshops

Findings from the systematic reviews and the formative work (phase I) will be shared during a participatory consultation workshop with 8–10 key stakeholders (stroke survivors, primary caregivers, health professionals, and disability and rehabilitation experts) who will be selected for the study. Such workshops will be conducted at the end of each phase of the research (3-workshops in total). The purpose of the workshop is to facilitate triangulation of the information obtained from each phase and to reach a decision on the best content for the intervention. The participatory consultation workshops will be organised to bring in the key stakeholders together to seek their opinions, extract their knowledge and to decide on the best content for the intervention in a collaborative and creative environment.

### Integrating the content of the education intervention with the Smartphone

The educational intervention developed during the formative phase of the research will be transformed into a Smartphone-enabled intervention. This process will enable stroke survivors and their caregivers to use a Smartphone to access the intervention. The steps involved in developing the Smartphone enabled education programme are as follows

1. The content of the stroke education intervention (eg, positioning techniques, pressure relief procedures, self-care tasks, functional ambulation and exercises) will be converted into an animated or an illustrated (using patient demonstration) video version.
2. The digitised animated/video version of the stroke education intervention will be uploaded onto a Smartphone using an appropriate (Android/Windows) application platform. This will enable the participants to access the educational intervention package using the Smartphone. If there are any operation problems in uploading or technical issues with the Smartphone application, the digitised video clips will be transferred onto a specific folder that contains videos in the Smartphone.
3. Once the stroke education intervention is uploaded onto the Smartphone, the Smartphone-enabled educational intervention will be ready for use by the participants.

## Phase II methods

### Phase II: Pre-testing of the intervention and stakeholder consultation

#### Field testing of the intervention

The provisional Smartphone-enabled intervention package will be field tested with a subsample of 30 adult stroke survivors and their caregivers. For this, a subsample of stroke survivors and their caregivers will be purposively identified from phase 1 survey respondents, excluding those who were part of the in-depth interview process. The Smartphone loaded with the intervention will be provided to the participants to be used at home for 2 weeks. Primary caregivers of stroke survivors selected for this phase will be asked to support the stroke survivors in accessing the intervention from the Smartphone.

#### Direct observation during field-testing

Utilisation of the Smartphone-enabled intervention and the support provided by the caregivers to the stroke survivors will be assessed by an Occupational Therapist (SK) using direct observation techniques during this phase. The main purpose of using a direct observation technique in this phase is to triangulate and affirm the information provided by the participants during phase 1. Some of the key issues that will be assessed during the direct observation include:

- Relevance and comprehensibility of the intervention;
- Operational difficulties of the participants in using the Smartphone;
- User-friendliness of the intervention;
- Technical issues in the Smartphone;
- Training needs in order to access the intervention from the Smartphone.

An observation checklist will be developed and used to assess these key issues during field-testing. The outcome of the field-testing will inform the development of a finalised version of a completely illustrated, pictorial training manual (user-friendly even for participants with low literacy level) explaining the operation of the Smartphone to access the intervention.

#### Stakeholder consultation: refinement of the educational intervention

The outcomes of the field testing phase will be shared with the key stakeholders for their feedback and recommendations during the second participatory consultation workshop. The objective of this workshop is to consult with stakeholders about the feasibility of the intervention, receive feedback and refine the intervention, as recommended by the stakeholders. The consultation workshop will be a participatory process as described above. Recommendations from the stakeholder consultations will be used to refine the intervention package for the pilot phase.

### Phase III: Piloting of the intervention and assessment of feasibility and acceptability

The objective of this pilot phase is to implement the intervention, and evaluate the feasibility and acceptability of the intervention. This phase will be carried out as a pilot study, which will provide useful information to plan a large scale RCT of the intervention in the future.

## Phase III: Methods

### Participants and eligibility criteria

Participants with a primary diagnosis of stroke will be recruited from VHS hospital in Chennai, India.

The inclusion criteria will be:

- Adults ≥18 years;
- Recent diagnosis of first ever stroke—as defined by the WHO^31^ within 3–6 weeks prior to the recruitment;
- Severity of stroke, mild and moderate (score 1–15, according to the NIH stroke scale);^32–34^
- Stroke survivor medically stable (reaching a point in medical treatment where life-threatening problems following stroke have been brought under control);
- Post-stroke functional status of the stroke survivor: requiring assistance of one person to perform basic activities of daily living including transfers, self-care and mobility;
- Stroke survivor residing with a primary caregiver (family member) at home.

The exclusion criteria will be

- Participants with NIH score >15;
- Severe cognitive difficulties (NIH stroke scale components for cognition);^34^
- Severe communication problem;
- Severe comorbidities (severe psychiatric illness, hearing loss, vision loss);
- Stroke survivor functionally dependent due to pre-existing conditions;
- Stroke survivor who does not have a primary caregiver;
- Stroke survivors who are unwilling/unable to adhere to the study protocol;
- Participants who do not qualify the training requirements (operation of Smartphone).

Eligibility assessment using NIH stroke scale will be conducted by the investigator to identify participants to be recruited for this pilot study.

### Participant recruitment for the pilot study

Participants for the pilot study will be recruited after their hospital discharge. Information about participants who are discharged from the hospital (in the past 3–6 weeks) will be retrieved from hospital records. An eligibility assessment will be completed within 2 weeks after identification of the participant from the hospital records. Participants identified for the piloting will be identified and contacted by phone. They will be informed about the purpose and processes of the study. If a participant is interested, written informed consent from the participant will be obtained in person. Consent procedures will be completed at the participant's home.

### Participants for the pilot study

A total of 30–40 participants will be recruited from VHS hospital in Chennai for the pilot phase. The admission rate of stroke survivors in this hospital is 3–4/weeks. Given the hospital admission rate, it will take 4–5 months to recruit 30–40 participants who will meet the eligibility criteria for this phase.

### Intervention procedure

The Smartphone uploaded with the intervention will be provided to the participants and we will show the stroke survivors and their caregivers how to use the Smartphone-enabled intervention. If the stroke survivor requires assistance, their caregivers will be encouraged to support them in using the intervention. The participants will be introduced to the intervention during initial home-visit. A structured training session for the stroke survivors and their caregivers on using the Smartphone-enabled intervention will be provided. The structured training will include:

- Introduction to the Smartphone-enabled intervention.
- Accessing the educational intervention package using the smart-phone application.

The finalised manual for Smartphone operation to access the intervention will be used during this training. A copy of the finalised Smartphone operation manual will be provided to the study participants for use at home. An occupational therapist (SK) will also assess whether the participants are able to use the Smartphone application (hands-on) appropriately during the training. An errorless attempt to retrieve the required part of the intervention from the Smartphone for more than three attempts will be considered to be successful training.

After successful training, the Smartphone enabled educational intervention package will be provided to the participants for use at their home for the next 4 weeks. Participants will also be encouraged to contact the study leader (SK) if they have any concerns regarding Smartphone operation during these 4 weeks.

### Assessment of outcomes

The primary outcomes of the pilot phase will be the feasibility and acceptability of the intervention. In addition to these outcomes, assessment of outcomes relating to the extent of disability and independence in activities of daily living will be carried out using the Modified Rankin scale^37^ and Barthel Index,^38^ respectively. Assessment of these outcomes will inform the feasibility of using these outcome assessment tools for future trials of the intervention. Details of the outcome assessment of phase 3 are explained in table 2.

**Table 2:** Details of the outcome assessment for the pilot testing phase

Outcomes	Description
Feasibility	A list of indicators will be developed during the pilot phase of the research study to assess feasibility of the intervention. This will include
	*Feasibility of recruitment:*
	Time taken to recruit the proposed number of participants Proportion of eligible participants identified Proportion of participants who consented in relation to participants who are eligible Reasons for exclusion
	*Training:*
	Number of participants successfully trained from the number of participants recruited for training Time taken for training by participants in different age-group, stroke severity and other factors (eg, experience of using a Smartphone) Training needs of participants in different age-groups, stroke severity and other factors
	*Study processes:*
	An in-built mechanism will be configured onto the Smartphone application to monitor the use of the intervention by participants. These indicators include:
	Proportion of participants ever using the application Proportion of participants using the application every week Proportion of participants using the application every day Proportion of participants using it for more than 1 h Proportion of participants requiring carer support Proportion of participants and carers successfully trained in using the application Proportion of participants accessing specific contents from the intervention Proportion of participants contacting the trainer/investigator for support Proportion of participants adhering to study protocol Reasons for non-adherence
	*Follow-up:*
	Number of drop-outs Reasons for dropping out
Acceptability	During the follow-up at the end of 4 weeks, a patient experience assessment will be conducted in order to understand the reasons for adherence or non-adherence, using a small questionnaire that will be developed for this purpose, soon after the end of phase 2 (once the intervention is ready)
Functional outcomes	Extent of disability—Modified Rankin Scale Activities of daily living—Barthel Index

### Analysis plan for the pilot phase

STATA will be used for analysis of the data in the pilot phase. Outcomes measuring the difference in proportions will be analysed using the χ^2^ test or Fisher's exact test. Outcomes measuring the difference in means will be analysed using the paired student t test or Wilcoxon matched pairs signed rank-sum test. Multivariate analysis using logistic regression techniques will also be conducted. To adjust for the imbalances in baseline characteristics, stratified analysis will be conducted or Mantel-Haenzel method will be used in the analysis.

### Stakeholder workshop

The study findings will be shared during the final stakeholder workshop at the end of the pilot testing (phase 3).

### Expected outcomes of this research study

This study seeks to develop and evaluate a Smartphone-enabled carer-supported educational intervention for management of post-stroke disability in India. Empirical exploration of this strategy will provide information on pragmatic solutions required to address the growing needs due to stroke disability in India and in other resource constrained settings. This research will provide an opportunity to develop a patient-centred educational intervention for management of post-stroke disability that is relevant to the context of low- and middle-income countries. Findings from the research will also provide valuable information about the resources required to deliver such interventions in resource-constrained settings.
